# Robotic-assisted versus video-assisted thoracoscopic enucleation for esophageal leiomyoma: A propensity-weighted retrospective cohort study

**DOI:** 10.1016/j.xjon.2026.101931

**Published:** 2026-06-17

**Authors:** Hengxuan Xu, Weidong Hu

**Affiliations:** a Department of Thoracic Surgery, ZhongNan Hospital of Wuhan University, Wuhan, Hubei, China; b Second Clinical College, Wuhan University, Wuhan, Hubei, China

**Keywords:** enucleation, esophageal leiomyoma, inverse probability of treatment weighting, robotic-assisted thoracic surgery, video-assisted thoracoscopic surgery

## Abstract

**Objective:**

The study objective was to compare recovery, operative outcomes, and direct in-hospital costs between robotic-assisted thoracic surgery and video-assisted thoracoscopic surgery for minimally invasive enucleation of esophageal leiomyoma.

**Methods:**

This was a single-center retrospective cohort (January 2017 to September 2025). The primary end point was postoperative length of stay. Adjusted comparisons used stabilized inverse probability of treatment weighting from a prespecified propensity score model.

**Results:**

Sixty-one patients were included (23 robotic-assisted; 38 video-assisted). In propensity-weighted analyses (robotic-assisted thoracic surgery – video-assisted thoracoscopic surgery), robotic-assisted thoracic surgery was associated with shorter postoperative length of stay (−0.85 days [−1.53 to −0.16]; *P* = .015), earlier chest drain removal (−0.83 days [−1.30 to −0.35]; *P* < .001), and lower estimated blood loss (−31.51 mL [−41.02 to −22.00]; *P* < .001), whereas total length of stay was similar (−0.81 days [−2.19 to 0.57]; *P* = .253) and direct in-hospital cost was higher (15,895 Chinese yuan [13,126-18,664; *P* < .001). Adverse events were infrequent. Length of stay end points were interpreted within a standardized institutional pathway.

**Conclusions:**

In this small single-center cohort, robotic-assisted thoracic surgery was associated with shorter selected recovery milestones at higher direct in-hospital cost. These hypothesis-generating findings describe local trade-offs rather than definitive evidence of platform superiority or subgroup selection.

**Figure:**
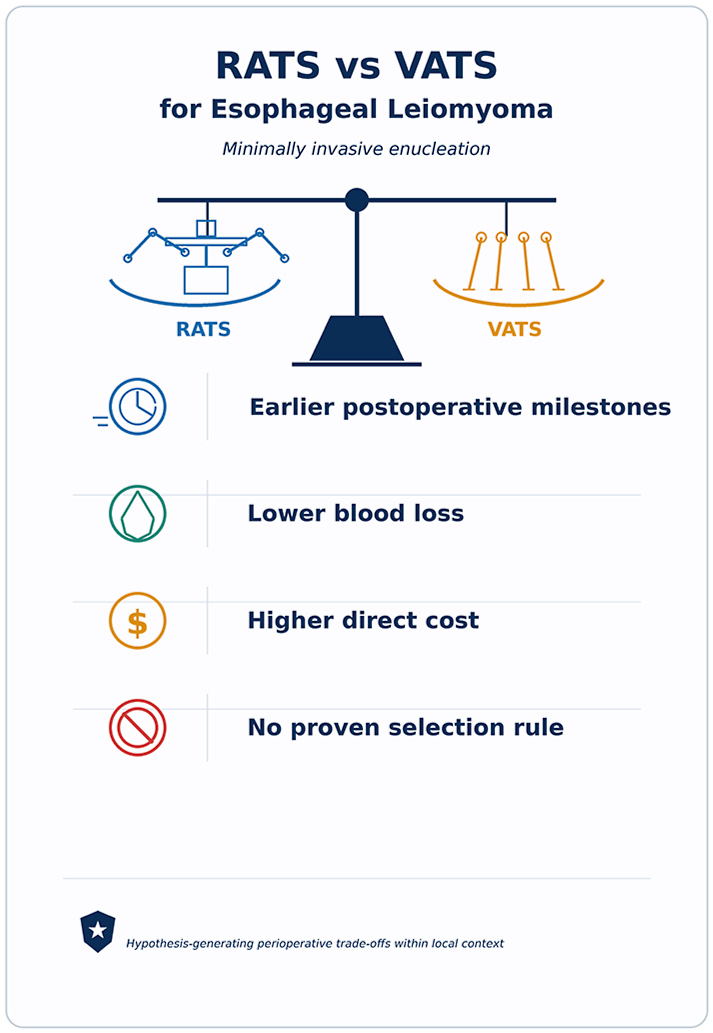
Hypothesis-generating perioperative trade-offs within local context.

**Central Message**

RATS was associated with shorter recovery milestones and lower blood loss than VATS, but at higher direct in-hospital cost; findings are hypothesis-generating.

**Perspective**

In benign esophageal leiomyoma, routine platform substitution may not be justified in all settings. Propensity-weighted analyses showed shorter recovery milestones and lower estimated blood loss with robotic surgery, but higher cost, framing local trade-offs rather than a complexity-based selection rule.

Esophageal leiomyoma is the most common benign mesenchymal tumor of the esophagus.^1^^,^^2^ Enucleation is generally considered the standard surgical approach for symptomatic tumors or lesions that enlarge over time,^3^, ^4^, ^5^, ^6^, ^7^, ^8^, ^9^ and minimally invasive techniques have largely replaced thoracotomy, with video-assisted thoracoscopic surgery (VATS) widely adopted for its reduced trauma and faster recovery^10^, ^11^, ^12^, ^13^

However, thoracoscopic enucleation can be technically demanding in the posterior mediastinum, where precise plane dissection and layered closure are required to preserve mucosal integrity and prevent pseudodiverticulum.^14^, ^15^, ^16^, ^17^ Robotic platforms offer 3-dimensional visualization and wristed instrumentation that may help reduce technical friction in constrained exposure and near-mucosal dissection,^18^, ^19^, ^20^ but whether these features justify higher costs for benign disease remains uncertain; comparative evidence remains limited and is largely derived from descriptive series.^13^^,^^16^^,^^21^ Accordingly, approach planning is often informed by analog experience from broader robotic thoracic procedures and minimally invasive esophageal surgery^22^, ^23^, ^24^

We performed a single-center retrospective cohort study using a prespecified propensity score/inverse probability of treatment weighting (IPTW) approach to estimate average treatment effects for recovery, operative outcomes, and costs after robotic-assisted thoracic surgery (RATS) versus VATS enucleation. We prioritized postoperative length of stay (PLOS) as the primary end point and interpreted the findings as perioperative trade-offs within a defined institutional pathway.

## Materials and Methods

### Study Design and Setting

The study was designed as a single-center retrospective cohort of consecutive patients undergoing minimally invasive thoracic enucleation for pathologically confirmed esophageal leiomyoma at ZhongNan Hospital of Wuhan University (January 2017 to September 2025). The study was approved by the Institutional Review Board of ZhongNan Hospital of Wuhan University (Approval No. 2026054K; 2/11/2026); informed consent was waived because deidentified retrospective data were used.

Inclusion criteria were pathologically confirmed leiomyoma treated by minimally invasive enucleation via RATS or VATS; exclusions included non-leiomyoma pathology (eg, gastrointestinal stromal tumor), concurrent major procedures, and missing key perioperative data. Patients were identified from surgical logs and pathology records; follow-up data came from charts and structured telephone follow-up. In our institutional workflow, preoperative evaluation typically included cross-sectional imaging and upper endoscopy, with endoscopic ultrasonography used selectively when lesion layer of origin or mucosal relationship required clarification; routine preoperative biopsy was not required before direct enucleation.

Because esophageal leiomyoma requiring minimally invasive enucleation is uncommon, the study size was determined by all eligible consecutive cases during the study period; no formal a priori power calculation was performed.

An a priori preoperative “high-complexity features” indicator was used for adjustment to capture anticipated technical difficulty (thoracic inlet/upper thoracic exposure constraints, proximity to critical structures, suspected mucosal adherence, or an indistinct submucosal plane). Features were adjudicated preoperatively during departmental case discussion by senior attending surgeons and the operating surgeon using pre-incision imaging/endoscopy. Tumor size 5 cm or more alone did not mandate high-complexity classification when computed tomography showed an accessible, well-circumscribed lesion with an expected clear dissection plane. Intraoperative events were not used for preoperative adjustment.

### Exposure and Perioperative Care

Procedures were performed by surgeons experienced in both platforms. Approach selection followed routine counseling and considered anticipated technical demands, platform availability/logistics, and patient preference when relevant; no formal patient-preference variable was available for adjustment. Enucleation followed standard principles: myotomy, submucosal-plane dissection, mucosal integrity testing, and myotomy closure. VATS was performed using a conventional multiport thoracoscopic approach; RATS was performed using a 3-port robotic thoracic configuration, with assistant access and port placement adjusted according to tumor level, side, and surgeon preference. A standardized perioperative pathway (multimodal analgesia, early mobilization, chest drain management, and diet advancement after leak exclusion) limited nonclinical variation in LOS-related end points. Follow-up used clinic visits and structured telephone assessment.

### Outcomes

The prespecified primary end point was PLOS (days from surgery to discharge). Secondary end points included total LOS (TLOS; admission to discharge), operative time (skin incision to chest closure), estimated blood loss, time to chest drain removal, pain duration, time to full recovery, complications (Clavien–Dindo), and direct in-hospital cost (Chinese yuan [CNY]). In our pathway, patients were commonly admitted before surgery for perioperative assessment, anesthesia review, insurance- or payment-policy-related inpatient testing, and operating-room scheduling; TLOS therefore includes preoperative inpatient time and was retained as a contextual hospital-use end point. PLOS was prioritized as the more direct recovery-related end point. Esophageal mucosal perforation recognized intraoperatively and repaired was recorded under postoperative complications because it breached esophageal integrity and prompted postoperative surveillance. Pain duration was days from surgery until no incisional/chest pain requiring analgesics for usual daily activity; full recovery was return to usual daily activities with tolerance of a normal oral diet.

### Statistical Analysis

The primary estimate was the average treatment effect, estimated using a prespecified logistic propensity-score model with stabilized IPTW; weights were capped at the first/99th percentiles. Weighted least-squares models with HC1-robust standard errors were used for continuous outcomes. Balance and overlap diagnostics (target |standardized mean difference| <.10) and prespecified sensitivity analyses (multivariable regression, augmented inverse probability weighting, and exploratory matching) are reported in the Supplement. Secondary outcomes were interpreted with attention to effect sizes and confidence intervals (no multiplicity adjustment).

Continuous variables are summarized as mean ± SD and categorical variables as n (%). Treatment effects are reported as mean differences (RATS–VATS) with 95% CIs.

To reduce confounding by indication, the primary propensity score model included only preoperative factors available before approach selection—body mass index, tumor size and location, comorbidity burden, symptomatic status, and the prespecified high-complexity indicator (Appendix E2, Table A1). An expanded sensitivity model additionally included age and sex. Stabilized IPTW weights were examined for extremes, winsorized at the first/99th percentiles, and balance was assessed with standardized mean differences (target <.10) and distributional checks.

After exclusions, core baseline covariates and all outcomes used in the primary analyses were complete under prespecified coding rules; primary models therefore used complete-case analysis without imputation. Outcome models used robust (sandwich) standard errors to account for weighting. Patients excluded for loss to follow-up lacked postdischarge recovery metrics (pain duration and time to full recovery), which were prespecified secondary end points; in-hospital end points used for primary analyses were otherwise complete.

Additional diagnostics, subgroup analyses, and sensitivity analyses are provided in Appendix E1 (Figure E1, Figure E2, Figure E3, Figure E4, Figure E5, Figure E6, Figure E7, Figure E8, Figure E9, Figure E10, Figure E11, Figure E12, Figure E13; Table E1, Table E2, Table E3, Table E4, Table E5, Table E6, Table E7) and Appendix E2 (Table A1, Table A10, Table A11, Table A1a, Table A2, Table A2b, Table A3, Table A4, Table A5, Table A6, Table A7, Table A8, Table A9).

## Results

**Figure 1:**
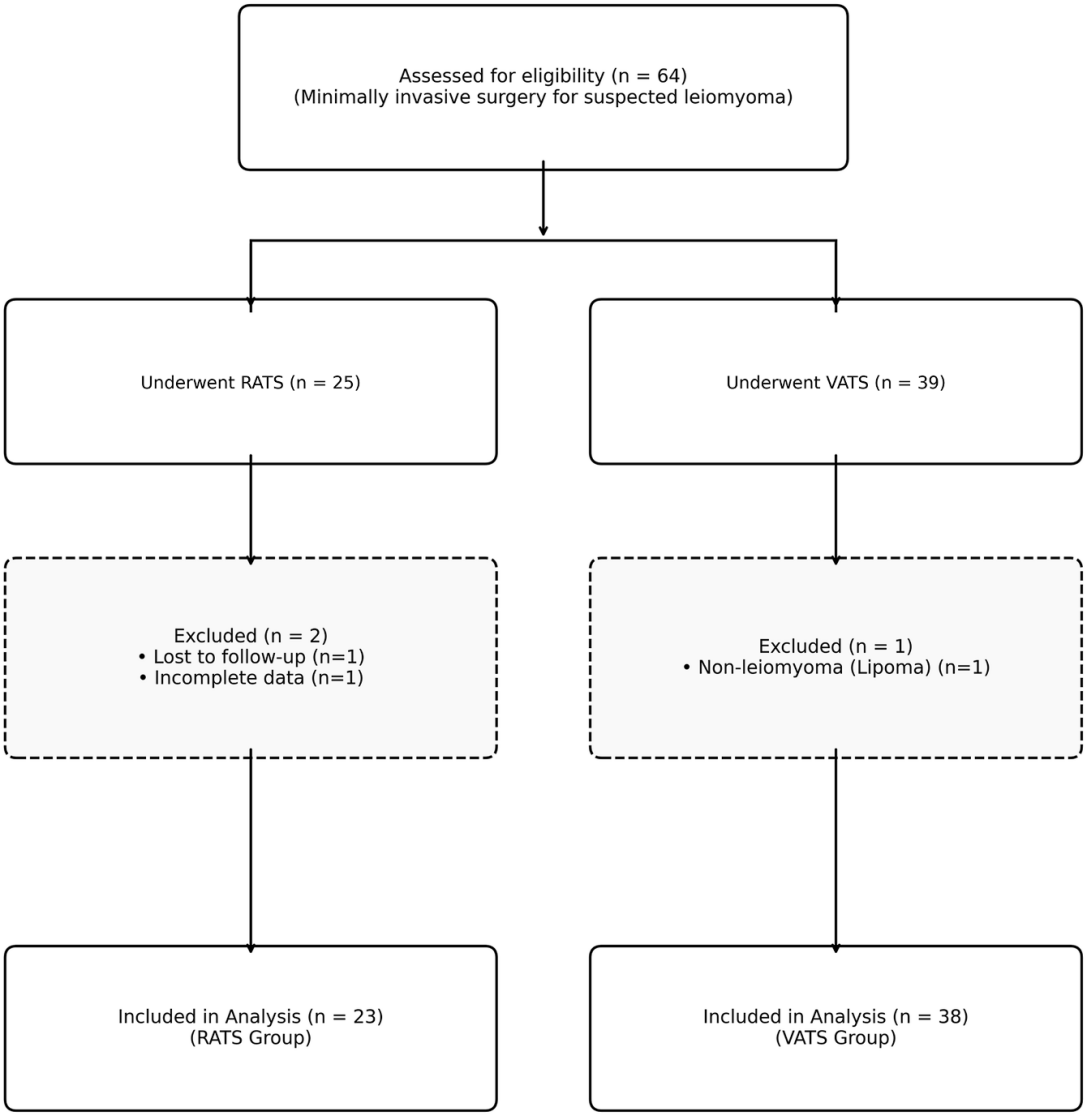
Study flow diagram (STROBE-style) of cohort assembly and exclusions. *RATS*, Robotic-assisted thoracic surgery; *VATS*, video-assisted thoracoscopic surgery.

Of 64 consecutive patients undergoing minimally invasive enucleation for suspected esophageal submucosal tumors, 61 with pathologically confirmed leiomyoma were included (RATS n = 23; VATS n = 38) (Figure 1). Treatment effects are reported as mean differences (RATS–VATS); negative values indicate lower values with RATS.

Baseline characteristics were broadly comparable (Table 1). After stabilized-weight IPTW, covariate balance improved substantially (Table E1); the high-complexity indicator showed a small residual imbalance (standardized mean difference| ≈ .12) and was therefore interpreted conservatively in sensitivity analyses.

**Table 1:** Baseline characteristics of the cohort

Characteristic	RATS (n = 23)	VATS (n = 38)	|SMD|
Age (y)	59.1 ± 16.8	55.9 ± 14.5	0.206
Sex			
Male	12 (52.2%)	21 (55.3%)	0.062
Female	11 (47.8%)	17 (44.7%)	0.062
BMI (kg/m^2^)	22.9 ± 3.4	22.9 ± 2.4	0.011
Any comorbidity	10 (43.5%)	9 (23.7%)	0.429
Symptomatic	19 (82.6%)	30 (78.9%)	0.093
Tumor size (cm)	4.6 ± 2.8	3.8 ± 2.1	0.322
Tumor location (cm from incisors)	28.4 ± 6.0	27.9 ± 4.5	0.089
Tumor location segment			
Upper (<25 cm)	5 (21.7%)	9 (23.7%)	0.046
Middle (25-30 cm)	10 (43.5%)	18 (47.4%)	0.078
Lower (>30 cm)	8 (34.8%)	11 (28.9%)	0.125
High-complexity features	3 (13.0%)	7 (18.4%)	0.148

*RATS*, Robotic-assisted thoracic surgery; *SMD*, standardized mean deviation; *VATS*, video-assisted thoracoscopic surgery.

In IPTW analyses (Table 2), RATS was associated with shorter PLOS (−0.85 (−1.53 to −0.16); *P* = .015), earlier drain removal (−0.83 (−1.30 to −0.35); *P* < .001), and lower estimated blood loss (−31.51 (−41.02 to −22.00); *P* < .001), while TLOS was similar (−0.81 (−2.19 to 0.57); *P* = .253) and direct in-hospital cost was higher (15,895 (13,126 to 18,664); *P* < .001).

**Table 2:** Unadjusted and inverse probability of treatment weighting–adjusted mean differences (robotic-assisted thoracic surgery– video-assisted thoracoscopic surgery) for continuous outcomes

Outcome	RATS (n = 23)	VATS (n = 38)	Unadjusted MD (95% CI)	IPTW MD (95% CI)	*P* value	Robustness
PLOS, d	4.2 ± 1.0	4.8 ± 1.3	−0.62 (−1.21 to −0.04)	−0.85 (−1.53 to −0.16)	.015	MV (HC1): *P* = .007
TLOS, d	8.6 ± 2.1	9.0 ± 2.7	−0.39 (−1.61 to 0.83)	−0.81 (−2.19 to 0.57)	.253	MV (HC1): *P* = .238
Estimated blood loss, mL	20.3 ± 12.1	46.9 ± 20.5	−26.61 (−34.97 to −18.25)	−31.51 (−41.02 to −22.00)	<.001	MV (HC1): *P* < .001
Operative time, min	71.4 ± 7.9	77.6 ± 9.1	−6.19 (−10.62 to −1.75)	−6.76 (−11.57 to −1.96)	.006	MV (HC1): *P* = .002
Time to chest drain removal, d	3.3 ± 0.7	3.9 ± 1.0	−0.63 (−1.08 to −0.19)	−0.83 (−1.30 to −0.35)	<.001	MV (HC1): *P* < .001
Postoperative pain duration, d	2.9 ± 1.1	4.2 ± 1.2	−1.27 (−1.88 to −0.66)	−1.49 (−2.19 to −0.79)	<.001	MV (HC1): *P* < .001
Time to full recovery, d	32.5 ± 6.2	37.1 ± 8.2	−4.61 (−8.34 to −0.88)	−6.95 (−11.21 to −2.70)	.001	MV (HC1): *P* < .001
Direct in-hospital cost, CNY	87,911 ± 4759	70,609 ± 4667	17,302 (14,789-19,814)	15,895 (13,126-18,664)	<.001	MV (HC1): *P* < .001

Mean differences are reported as RATS–VATS; negative values indicate lower values with RATS, whereas positive values indicate higher values with RATS. IPTW estimates used stabilized weights capped at the 1st/99th percentiles with HC1 robust standard errors. *RATS*, Robotic-assisted thoracic surgery; *VATS*, video-assisted thoracoscopic surgery; *MD*, mean difference; *IPTW*, inverse probability of treatment weighting; *CNY*, Chinese yuan; *MV*, multivariable.

**Figure 2:**
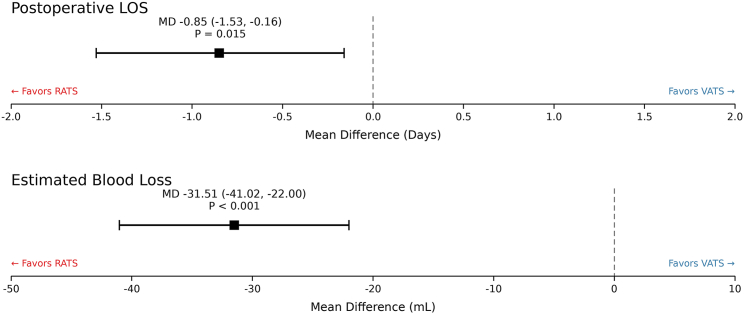
IPTW mean differences for PLOS and estimated blood loss. Points and 95% CIs are shown; the *dashed line* indicates no difference. Mean differences are RATS–VATS; negative values indicate lower values with RATS.

Figure 2 visualizes the primary IPTW effect estimates for PLOS and estimated blood loss.

Secondary recovery end points (pain duration and time to full recovery) were also lower in RATS, whereas operative time differences were modest (Table 2). Exploratory visualizations and additional sensitivity analyses are provided in the Supplement.

In IPTW analyses, the −0.85-day PLOS reduction for RATS (Table 2) was accompanied by earlier chest drain removal (−0.83 days), consistent with aligned pathway milestones. By contrast, TLOS was similar between groups, consistent with institutional practice in which preoperative inpatient evaluation and local payment/scheduling workflows may occur before surgery regardless of approach; thus, admission-to-discharge LOS may reflect local logistics even when postoperative recovery differs.

Unadjusted mean differences were directionally consistent with the IPTW estimates across the prespecified end points (Table 2), suggesting that weighting improved comparability rather than reversing the qualitative pattern. Lower values in RATS were also observed for pain duration and time to full recovery.

Operative efficiency metrics showed only modest between-group differences. Lower estimated blood loss with RATS (−31.51 mL) is small in absolute terms for leiomyoma enucleation but may be consistent with smoother plane-based dissection and hemostasis during confined mediastinal work. Exploratory visualizations, including size-stratified patterns, and additional robustness checks are provided in the Supplement.

Although RATS was associated with higher direct in-hospital cost (+15,895 CNY), this difference occurred alongside shorter postoperative stay and earlier drain removal and is interpreted here as a value trade-off rather than a direct measure of efficiency.

Adverse events and postoperative complications were infrequent and are summarized descriptively (Table 3); because event counts were sparse, the study was not powered for comparative complication inference. Overall adverse events occurred in 3/23 (13.0%) after RATS and 9/38 (23.7%) after VATS.

**Table 3:** Intraoperative and postoperative adverse events and Clavien–Dindo grades

Outcome	RATS (n = 23)	VATS (n = 38)	*P* value
Any adverse event (intraoperative or postoperative)	3 (13.0%)	9 (23.7%)	.508
Intraoperative adverse events			
Any intraoperative adverse event	0 (0.0%)	1 (2.6%)	1.000
Tracheal injury requiring repair	0 (0.0%)	1 (2.6%)	1.000
Bleeding requiring transfusion	0 (0.0%)	0 (0.0%)	Not applicable
Conversion to thoracotomy	0 (0.0%)	0 (0.0%)	Not applicable
Postoperative complications			
Any postoperative complication	3 (13.0%)	8 (21.1%)	.511
Esophageal mucosal perforation (repaired intraoperatively)	1 (4.3%)	2 (5.3%)	1.000
Pneumonia	1 (4.3%)	1 (2.6%)	1.000
Recurrent laryngeal nerve injury/hoarseness	0 (0.0%)	2 (5.3%)	.522
Chylothorax	0 (0.0%)	1 (2.6%)	1.000
Gastroesophageal reflux	1 (4.3%)	2 (5.3%)	1.000
Clavien–Dindo classification (postoperative complications only; consensus-adjudicated summary)			
Any postoperative complication with Clavien grade (≥I)	3 (13.0%)	8 (21.1%)	.511
Grade I	0 (0.0%)	3 (7.9%)	.284
Grade II	3 (13.0%)	5 (13.2%)	1.000
Grade ≥III	0 (0.0%)	0 (0.0%)	Not applicable

*P* values are descriptive Fisher exact tests because event counts were sparse; the study was not powered for comparative complication inference. *RATS*, Robotic-assisted thoracic surgery; *VATS*, video-assisted thoracoscopic surgery.

No conversions to thoracotomy or transfusions occurred; one tracheal injury required repair, and no postoperative complication reached Clavien–Dindo grade ≥III. Most graded events were Clavien–Dindo II and managed medically; mucosal perforation repaired intraoperatively remained rare and was captured under postoperative complications. Direct in-hospital cost was higher with RATS in propensity-weighted analyses (+15,895 CNY; 95% CI, 13,126-18,664; *P* < .001; Table 2), contrasting with recovery end points. A calendar-time sensitivity analysis adjusting for surgery year yielded directionally similar estimates (Appendix E2, Table A11).

## Discussion

**Figure 3:**
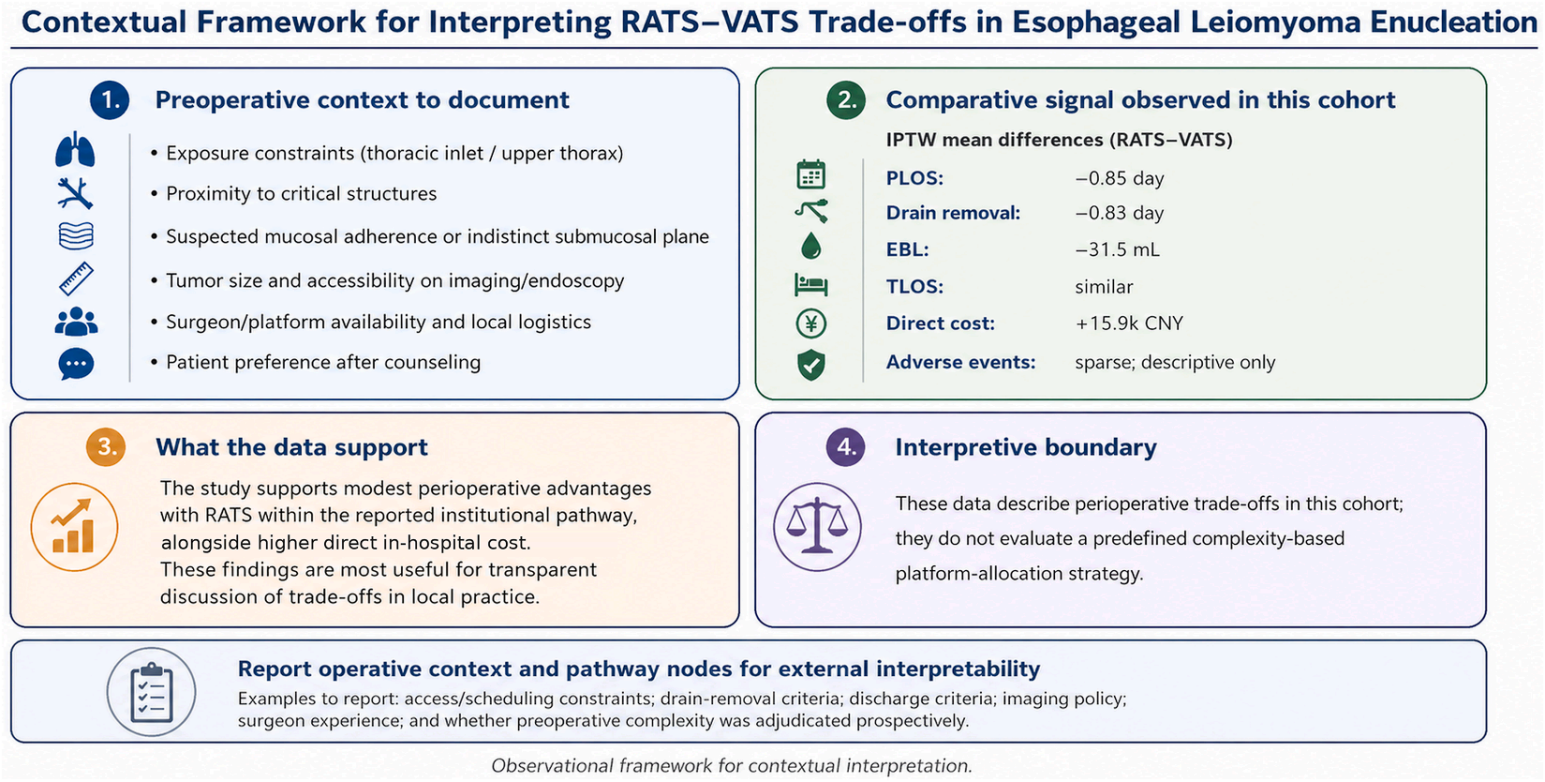
Contextual framework for RATS versus VATS trade-offs in minimally invasive enucleation. The figure summarizes preoperative planning factors, perioperative associations, and local technical/economic context. It supports transparent interpretation rather than a platform-selection algorithm. *RATS*, Robotic-assisted thoracic surgery; *VATS*, video-assisted thoracoscopic surgery; *PLOS*, postoperative length of stay; *TLOS*, total length of stay; *EBL*, estimated blood loss; *CNY*, Chinese yuan.

In this small single-center retrospective cohort within a standardized pathway, RATS was associated with modestly shorter postoperative stay and earlier drain removal at higher direct in-hospital cost, without clear differences in TLOS or major adverse events. The study therefore describes hypothesis-generating local perioperative trade-offs within a defined pathway (Figure 3). These recovery gains should not be interpreted as demonstrating that the higher direct in-hospital cost was offset or recouped.

TLOS requires caution across health-care systems. Unlike health systems with same-day or near-same-day admission pathways, our pathway includes preoperative admission for assessment, anesthesia review, insurance/payment-policy-related inpatient testing, and operating-room scheduling. Thus, TLOS may reflect preoperative pathway factors, including operating-room assignment timing, rather than postoperative recovery alone. Because admission-to-operation intervals and delay reasons were not uniformly captured, these data do not show systematic waiting for robotic access; TLOS was not used to infer recovery, access, or scheduling efficiency.

Strengths include a prespecified stabilized-IPTW (average treatment effect) analysis with robust standard errors, transparent balance diagnostics, and multiple sensitivity analyses. However, residual confounding remains possible, and the small sample size limits precision for rare adverse events and supports hypothesis generation rather than definitive conclusions. Recovery milestones and costs are pathway- and setting-dependent (eg, drain criteria, bed availability, reimbursement, operating room logistics),^25^ and patient-reported recovery could not be blinded. The numerically higher frequency of recurrent laryngeal nerve injury, chylothorax, and tracheal injury in VATS should be viewed as hypothesis-generating.

## Conclusions

Future work should validate these findings in multicenter cohorts with explicit capture of technical adverse events, standardized pathway definitions, and formal cost-component analyses. Prospective registries or pragmatic studies that predefine pathway milestones and capture granular intraoperative events would further strengthen causal interpretation and external validity.

In this small single-center cohort of patients undergoing minimally invasive enucleation for esophageal leiomyoma, RATS was associated with shorter postoperative recovery milestones and lower blood loss, but higher cost. These findings are hypothesis-generating and pathway-dependent, not definitive evidence of platform superiority or generalizable selection. Larger multicenter studies should clarify the clinical and economic context for robotic assistance.

### Declaration of Generative AI and AI-Assisted Technologies in the Writing Process

During the preparation of this manuscript, the authors used ChatGPT (OpenAI) to assist with English grammar and phrasing checks. The tool was not used to generate scientific content, perform statistical analyses, or draw clinical conclusions. All outputs were reviewed and edited by the authors, who take full responsibility for the integrity and accuracy of the work.

### Data Sharing and Reproducible Research

Deidentified participant-level data and statistical code necessary to reproduce the analyses may be made available from the corresponding author on reasonable request, subject to institutional approval, applicable data-use agreements, and privacy/ethics restrictions.

## Conflict of Interest Statement

The authors reported no conflicts of interest.

The *Journal* policy requires editors and reviewers to disclose conflicts of interest and to decline handling or reviewing manuscripts for which they may have a conflict of interest. The editors and reviewers of this article have no conflicts of interest.

## Figures and Tables

**Figure E1:**
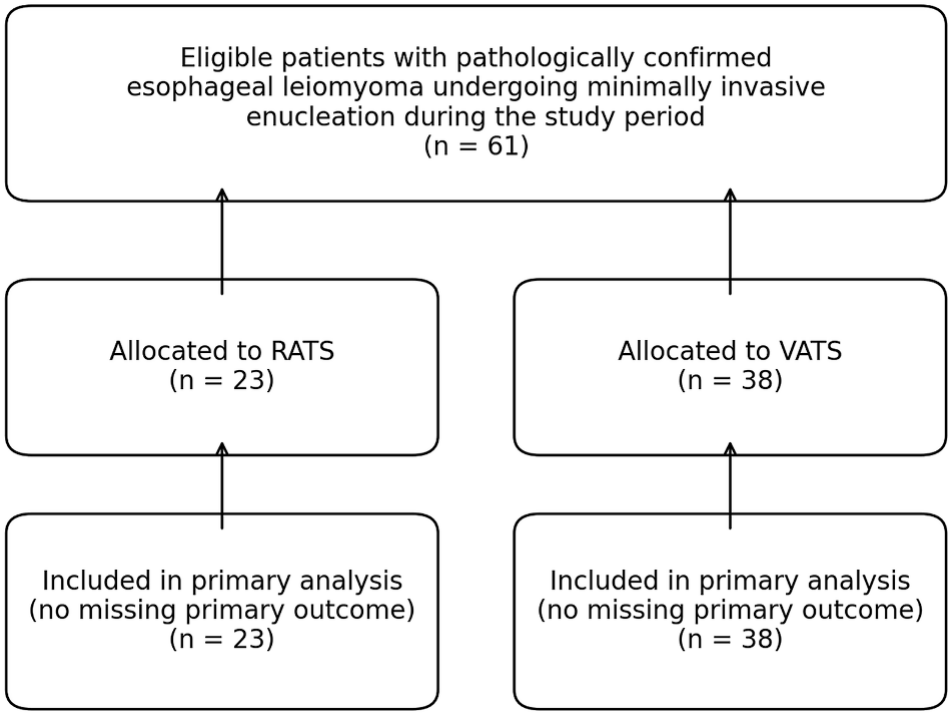
Study flow diagram of cohort inclusion and analysis population.

**Figure E2:**
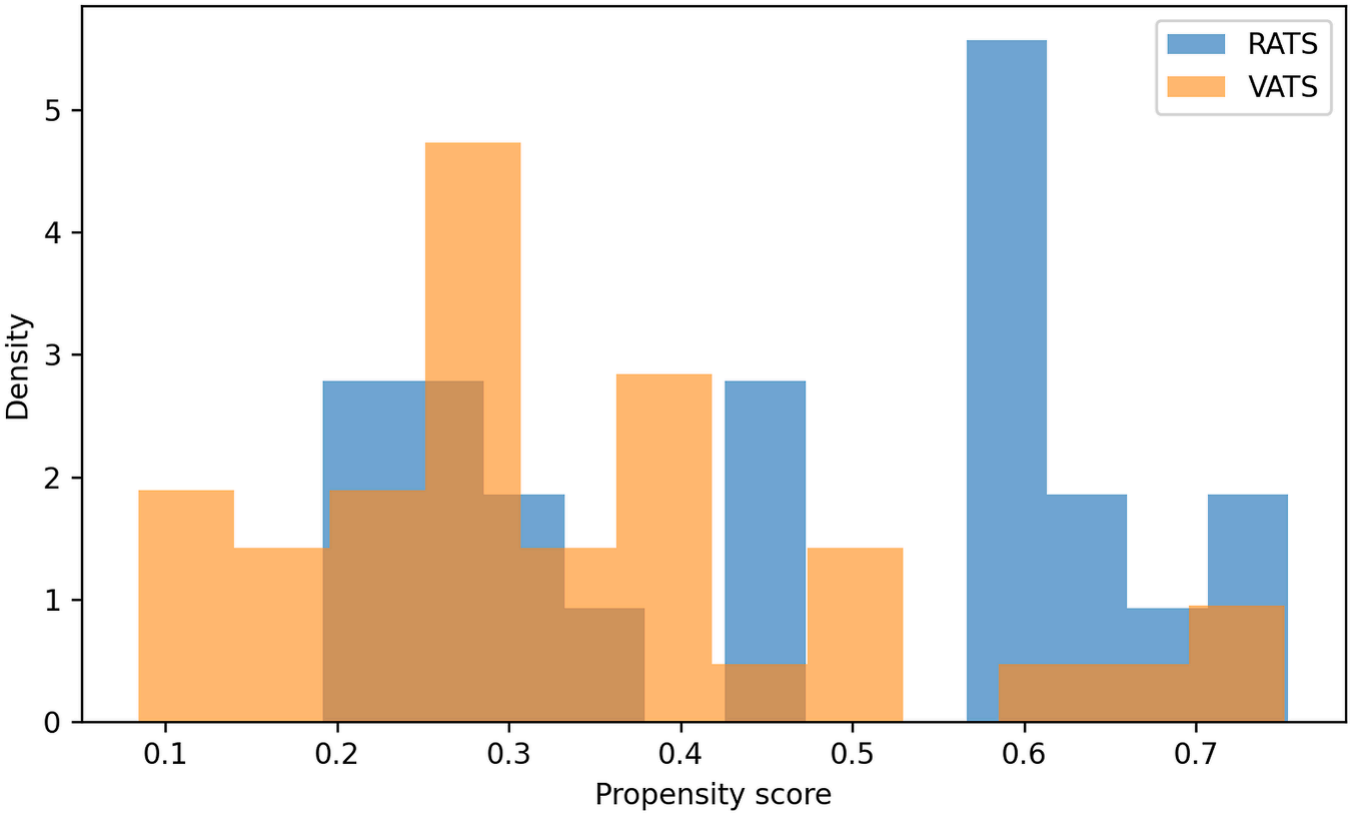
Propensity-score overlap supporting IPTW positivity.

**Figure E3:**
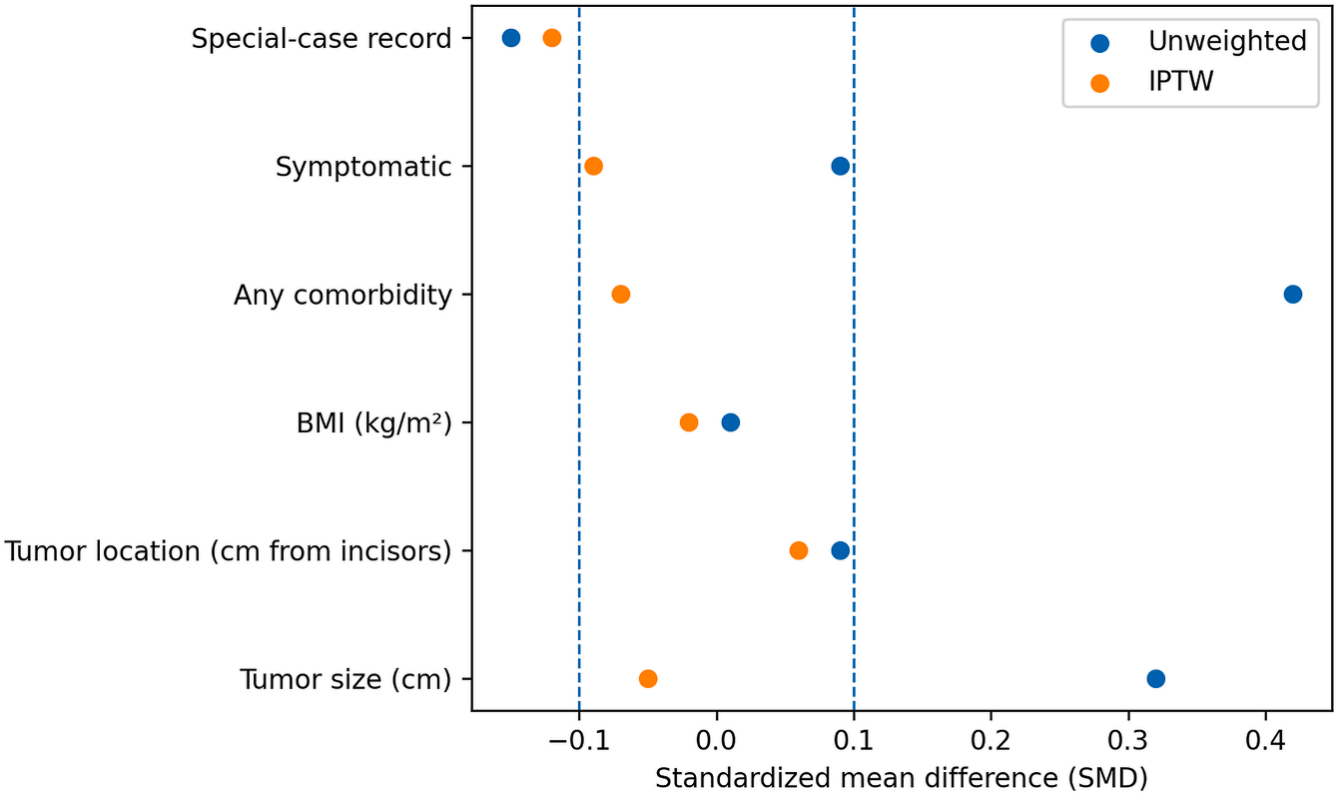
Covariate balance before and after IPTW.

**Figure E4:**
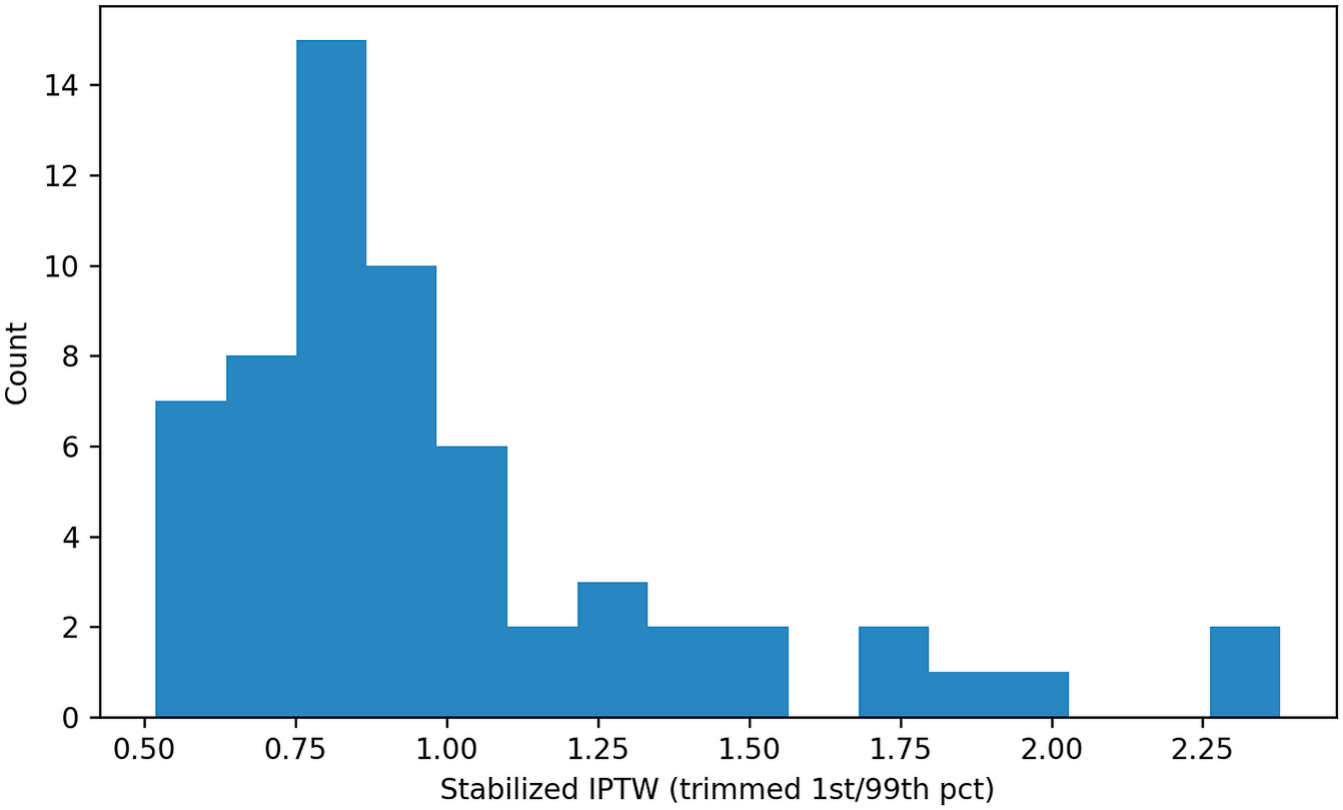
Stabilized IPTW weights after 1st/99th percentile winsorization.

**Figure E5:**
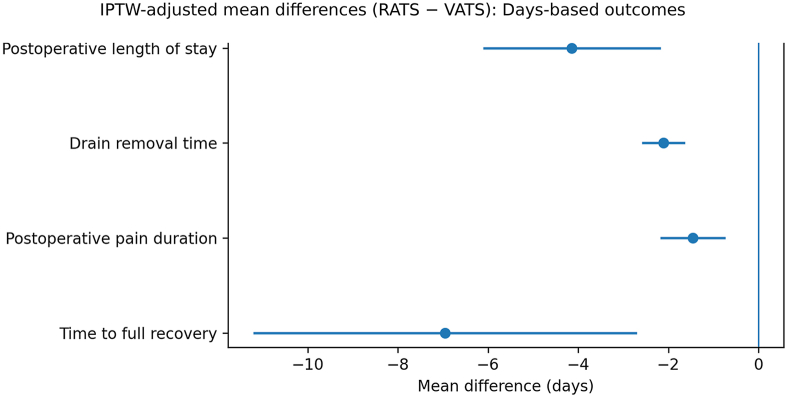
IPTW-adjusted mean differences (RATS–VATS) for days-based outcomes.

**Figure E6:**
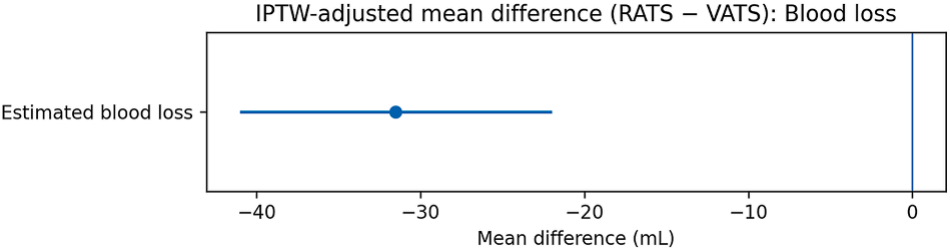
IPTW-adjusted mean difference (RATS–VATS) for estimated blood loss (mL).

**Figure E7:**
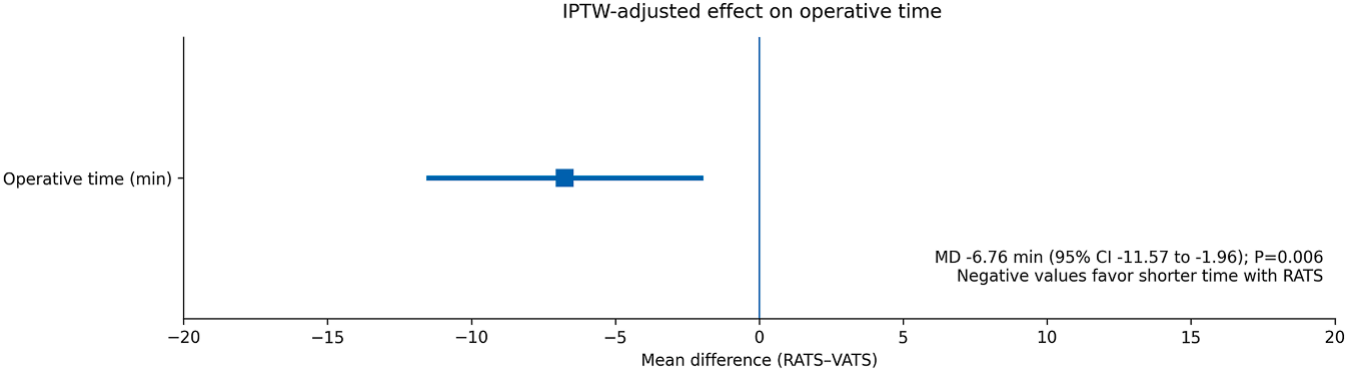
IPTW-adjusted mean difference (RATS–VATS) for operative time (min).

**Figure E8:**
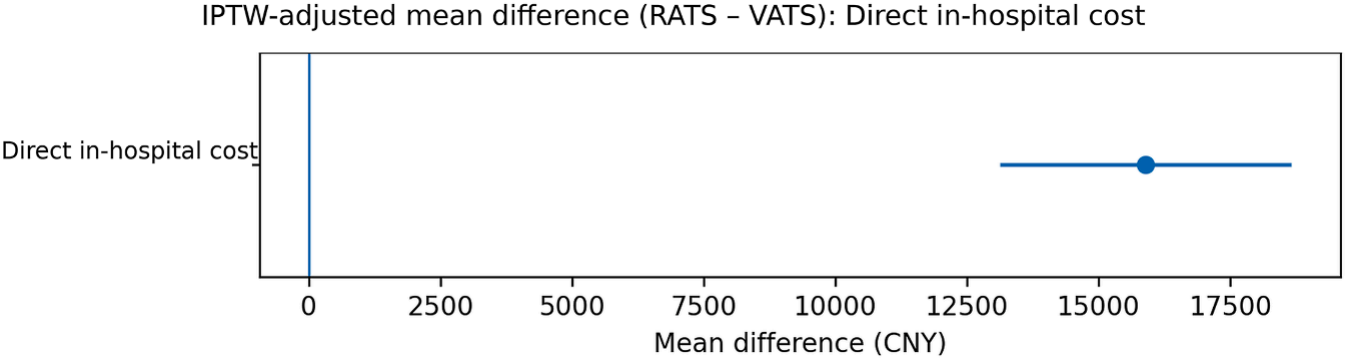
IPTW-adjusted mean difference (RATS–VATS) for direct in-hospital cost (CNY).

**Figure E9:**
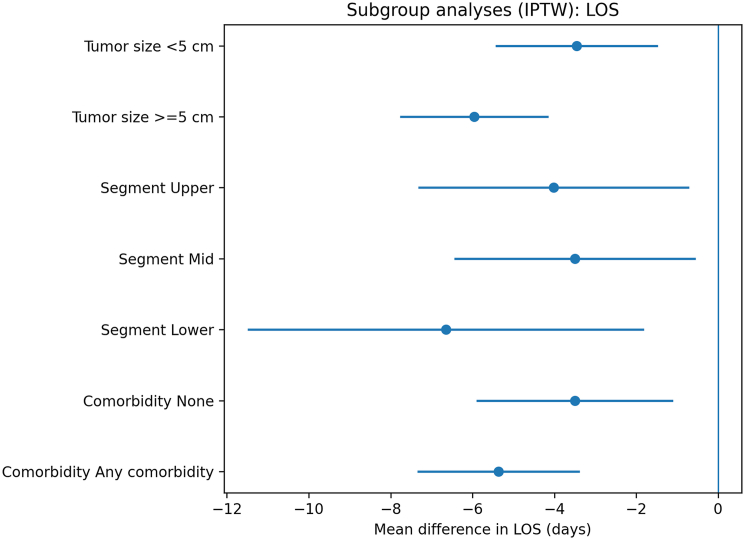
Subgroup-specific IPTW estimates for LOS.

**Figure E10:**
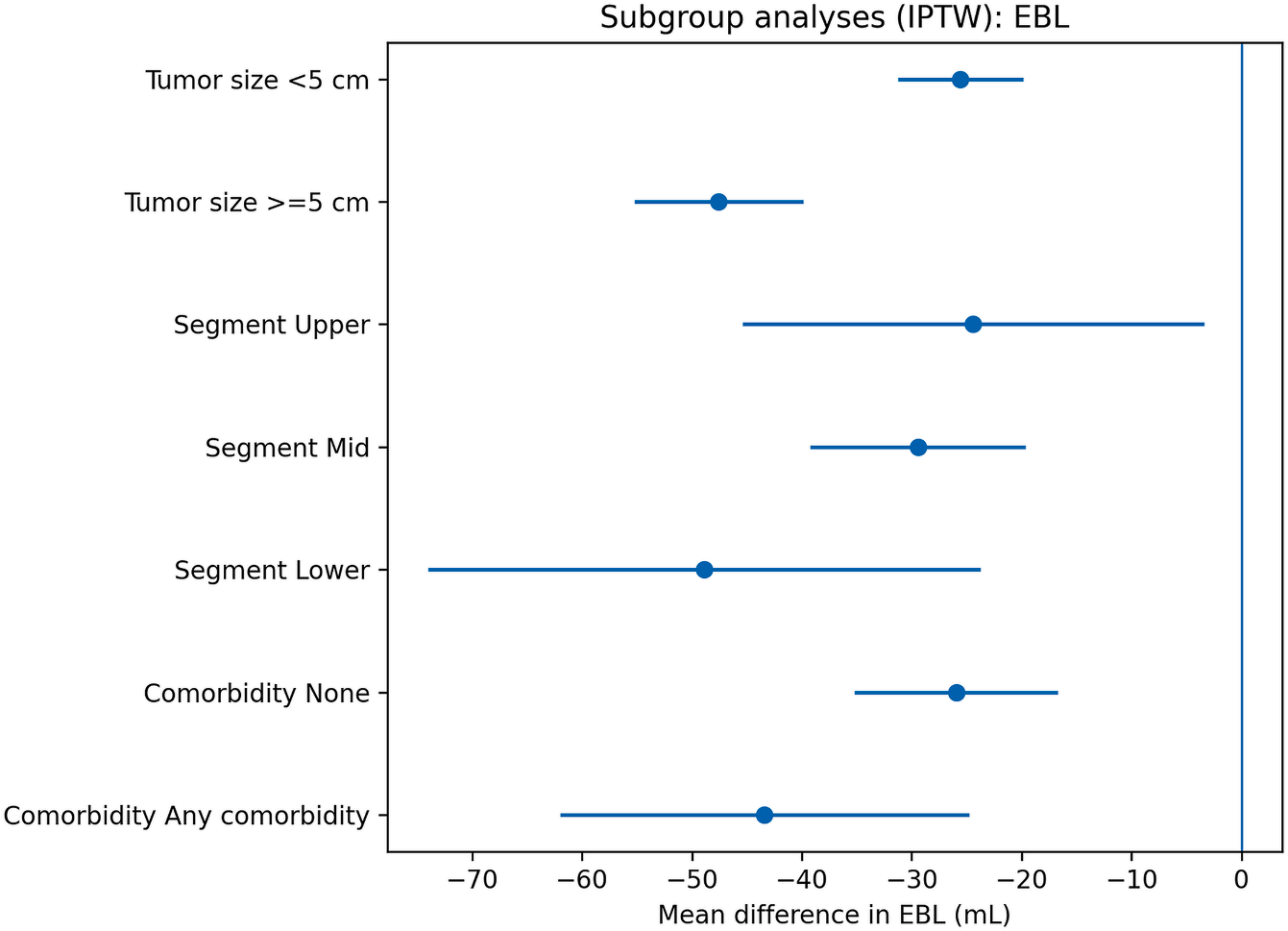
Subgroup-specific IPTW estimates for estimated blood loss.

**Figure E11:**
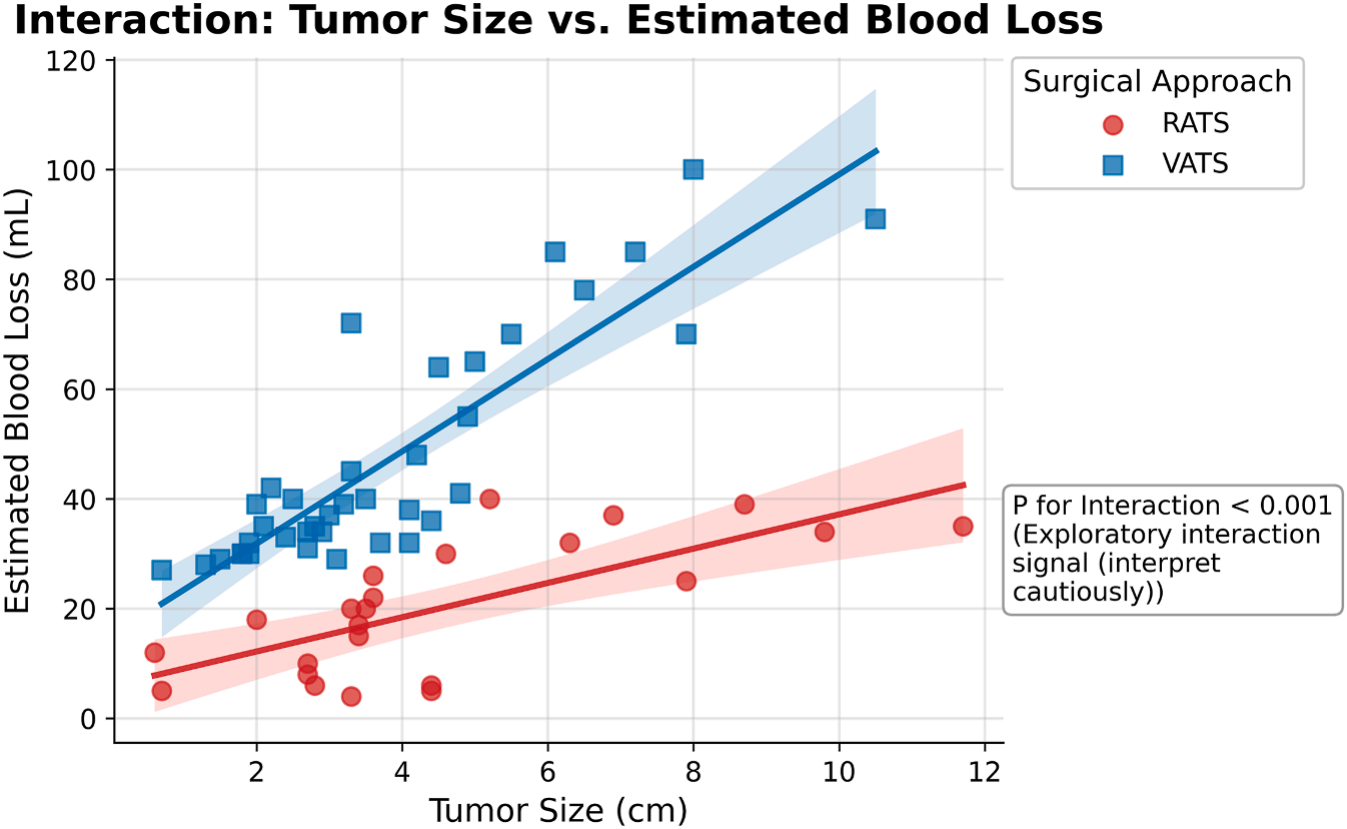
Tumor size versus estimated blood loss by approach (exploratory).

**Figure E12:**
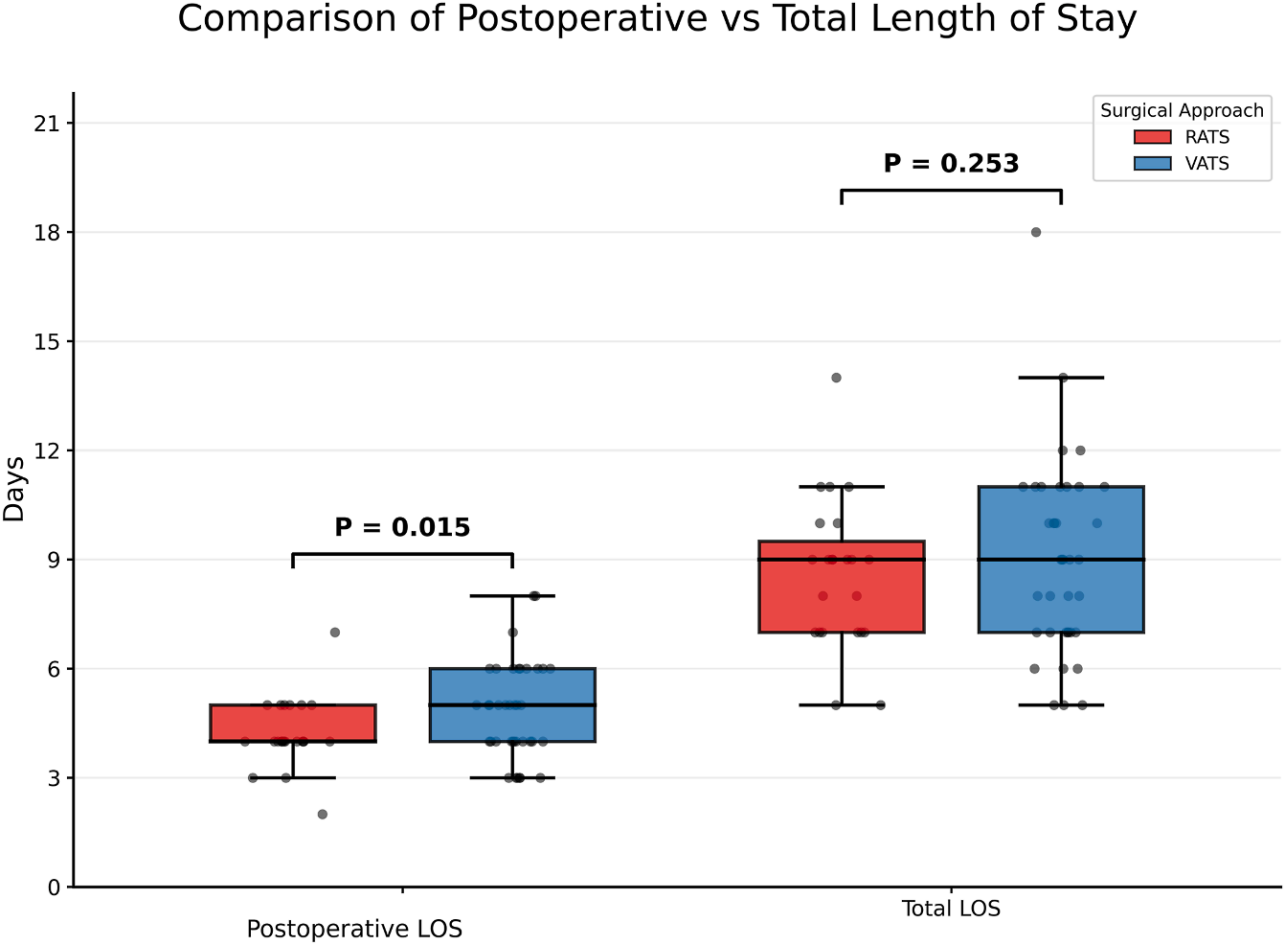
Distributions of PLOS and TLOS by approach (descriptive).

**Figure E13:**
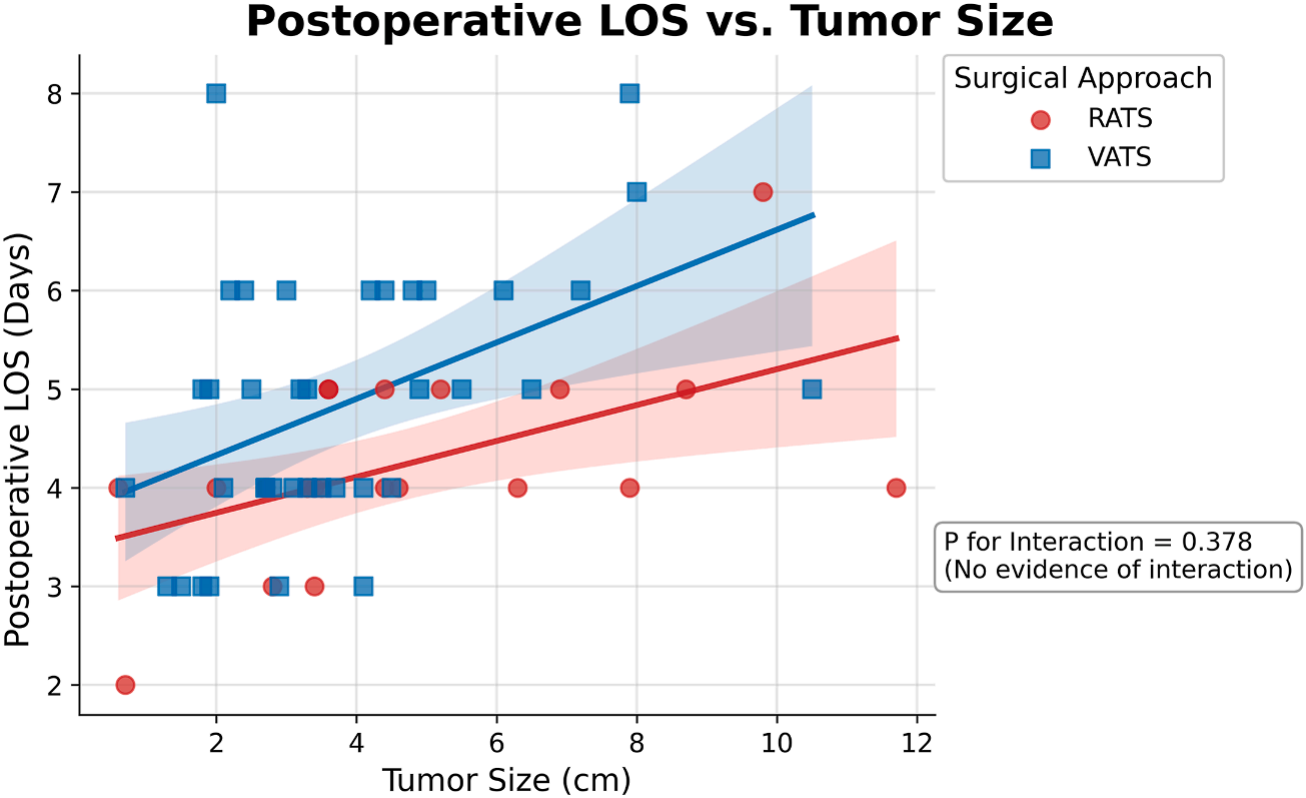
Tumor size versus PLOS by approach (exploratory).

**Table E1:** Covariate balance (standardized mean difference)

Covariate	|SMD| (unweighted)	|SMD| (IPTW)
Tumor size (cm)	0.32	0.05
Tumor location (cm from incisors)	0.09	0.06
BMI (kg/m^2^)	0.01	0.02
Any comorbidity	0.429	0.07
Symptomatic	0.09	0.09
High-complexity features record	0.15	0.12

Standardized mean differences quantify balance between groups; |SMD| < .10 is often used as a practical target. High-complexity features represent preoperative adjudication of anticipated technical difficulty and were not determined by tumor size alone; tumors ≥5 cm could be classified as noncomplex when anatomically accessible with well-circumscribed lesions and clear boundaries on CT (definitions in Appendix E2, Table A1). *IPTW*, Inverse probability of treatment weighting; *BMI*, body mass index; *SMD*, standardized mean difference.

**Table E2:** Effective sample size

Group	Observed N	Effective sample size
RATS	23	18.9
VATS	38	33.3
Overall	61	52.2

Effective sample size summarizes information retained under weighting; smaller ESS implies greater variance inflation. *RATS*, Robotic-assisted thoracic surgery; *VATS*, video-assisted thoracoscopic surgery.

**Table E3:** E-values (dichotomized outcomes)

Outcome	Threshold	IPTW or (95% CI)	E-value	E-value (CI limit)
Prolonged total LOS (TLOS > median)	>9 d	0.31 (0.10-1.00)	5.90	1.00
Delayed full recovery (>median)	>35 d	0.26 (0.09-0.81)	7.06	1.77

E-values are reported for dichotomized outcomes because the method is defined on the risk ratio/odds ratio scale. Prolonged total LOS was defined as TLOS greater than the cohort median (9 d), and delayed recovery as time to full recovery greater than the cohort median (35 d). *IPTW*, Inverse probability of treatment weighting; *LOS*, length of stay; *TLOS*, total length of stay.

**Table E4:** Extended breakdown of intraoperative adverse events and postoperative complications

Category	RATS (n = 23)	VATS (n = 38)	*P* value
Intraoperative adverse events			
Any intraoperative adverse event	0 (0.0%)	1 (2.6%)	1.000
Tracheal injury requiring repair	0 (0.0%)	1 (2.6%)	1.000
Bleeding requiring transfusion	0 (0.0%)	0 (0.0%)	Not applicable
Conversion to thoracotomy	0 (0.0%)	0 (0.0%)	Not applicable
Postoperative complications			
Any postoperative complication	3 (13.0%)	8 (21.1%)	.511
Esophageal mucosal perforation (repaired intraoperatively)	1 (4.3%)	2 (5.3%)	1.000
Pneumonia	1 (4.3%)	1 (2.6%)	1.000
Recurrent laryngeal nerve injury	0 (0.0%)	2 (5.3%)	.522
Chylothorax	0 (0.0%)	1 (2.6%)	1.000
Gastroesophageal reflux	1 (4.3%)	2 (5.3%)	1.000
Clavien–Dindo classification (postoperative only; highest per patient)			
Any grade (≥I)	3 (13.0%)	8 (21.1%)	.511
Grade I	0 (0.0%)	3 (7.9%)	.284
Grade II	3 (13.0%)	5 (13.2%)	1.000
Grade ≥III	0 (0.0%)	0 (0.0%)	Not applicable

Intraoperative adverse events and postoperative complications were tabulated separately. Clavien–Dindo grades were assigned only to postoperative complications, and the highest postoperative grade per patient is reported. Event timing/type and grading were adjudicated by consensus chart review. Counts are descriptive because events were sparse; Fisher exact *P* values should not be interpreted as evidence of equivalence or superiority. *RATS*, Robotic-assisted thoracic surgery; *VATS*, video-assisted thoracoscopic surgery.

**Table E5:** Covariate balance before and after propensity score matching (standardized mean difference)

Covariate	RATS (pre)	VATS (pre)	|SMD| (pre)	RATS (post)	VATS (post)	|SMD| (post)
Age (y)	59.13	55.89	0.206	55.88	57.94	0.128
Female sex	47.8%	44.7%	0.062	50.0%	43.8%	0.125
BMI (kg/m^2^)	22.90	22.87	0.011	23.11	22.59	0.188
Tumor location (cm from incisors)	28.39	27.92	0.089	29.75	28.88	0.207
Tumor size (cm)	4.59	3.79	0.322	4.22	3.99	0.089
Any comorbidity (yes)	43.5%	23.7%	0.429	25.0%	25.0%	0.000
Symptomatic (yes)	82.6%	78.9%	0.093	75.0%	62.5%	0.270
Preoperative high-complexity features (yes)	13.0%	18.4%	0.148	12.5%	6.2%	0.214

SMDs before and after 1:1 nearest-neighbor propensity score matching (PSM) without replacement using a caliper of 0.2 standard deviations of the logit propensity score. Sixteen matched pairs were retained. Continuous variables are summarized as means and binary variables as proportions. Absolute SMD values are shown; |SMD| < .10 is commonly used to indicate adequate balance. In this cohort, several covariates remained imbalanced after matching (|SMD| > .10), so PSM was interpreted as exploratory and not used as primary support. *RATS*, Robotic-assisted thoracic surgery; *VATS*, video-assisted thoracoscopic surgery; *SMD*, standardized mean difference; *BMI*, body mass index.

**Table E6:** Follow-up schedule and outcome ascertainment

Timepoint	Mode	Assessments	How used in this study
Postoperative wk 1 (POD 7 ± 3)	Outpatient clinic (study hospital)	Chest CT; complete blood count; basic metabolic profile (liver/renal function, glucose, electrolytes)	Assessed clinically relevant postoperative abnormalities; supported recovery assessment and complication ascertainment when available.
Postoperative mo 6	Outpatient clinic (study hospital)	Chest CT; complete blood count; basic metabolic profile (liver/renal function, glucose, electrolytes)	Surveillance for clinically meaningful abnormalities; corroborated complication status and recovery when available.
After mo 6 until 3 y postoperatively or December 31, 2025	Local hospital visits (patient choice) + telephone tracking	Imaging/labs per local practice (if performed); symptom review by phone	Post-discharge surveillance primarily via telephone; local test results were recorded when provided, but were not required to define full recovery.
Postdischarge mo 1, 6, 9, 12, 15, 18, 21, 24, 27, 30, 33, and 36	Structured telephone follow-up (scheduled timepoints; trained team; standardized script)	Standardized questionnaire: symptom status, pain severity and analgesic use, diet tolerance, daily activity level, and unresolved complications	Primary ascertainment of postoperative pain duration and time to full recovery; identical schedule across groups. Blinding to surgical approach was not feasible; these end points are supportive and interpreted cautiously.

Follow-up was continued for up to 3 years after surgery or until 31 December 2025, whichever occurred first. Clinic follow-up was typically performed at postoperative day 7 (±3 d) and at 6 months. Structured telephone follow-up was scheduled at 1 month after discharge and then at 6, 9, 12, 15, 18, 21, 24, 27, 30, 33, and 36 months using a standardized script by trained personnel; blinding was not feasible. Imaging and laboratory results were used to detect clinically significant postoperative abnormalities when obtained; however, their availability was not required to define full recovery. *POD*, Postoperative day.

**Table E7:** Key institutional pathway nodes and operational criteria relevant to length of stay

Pathway node	Operational definition in this center
Diet advancement	Stepwise oral intake as tolerated; additional studies/endoscopy performed only if clinically indicated (eg, symptoms or abnormal imaging).
Imaging policy	Routine chest CT on POD 2 to assess lung re-expansion and exclude pleural/mediastinal collections; additional imaging triggered by symptoms or abnormal labs.
Drain removal	Minimal, non-chylous and non-purulent output; no air leak; reassuring imaging and inflammatory indices; no clinical concern for ongoing complication.
Discharge	Typically, the day after drain removal if afebrile, ambulatory, tolerating oral intake, and pain controlled on oral analgesics without IV rescue; deferred if any criterion not met.

Pathway nodes are reported to contextualize LOS-related end points and enhance cross-center interpretability; they do not imply comparative performance of any specific pathway. Because pathway structures and discharge practices may vary across centers and over time, these nodes are provided for context rather than benchmarking or prescriptive recommendations. *CT*, Computed tomography; *POD*, postoperative day.

**Table A1:** Covariates and coding for propensity score models

Covariate	Type/coding	Primary PS model	Expanded PS sensitivity model
BMI (kg/m^2^)	Continuous	Yes	Yes
Tumor location (cm from incisors)	Continuous	Yes	Yes
Tumor size (cm)	Continuous	Yes	Yes
Any comorbidity	Binary: 1 if any listed comorbidity; 0 otherwise	Yes	Yes
Symptomatic status	Binary: 1 if any preoperative symptom reported; 0 if asymptomatic	Yes	Yes
Preoperative high-complexity features indicator	Binary: 1 if any prespecified high-complexity feature present (Table A1a); 0 otherwise; adjudicated preoperatively during departmental case discussions by senior attending surgeons together with the operating surgeon based on pre-incision imaging/endoscopy. Adjudication considered anatomic accessibility and the anticipated dissection plane; tumor size ≥ 5 cm alone did not automatically constitute high-complexity when lesions were well-circumscribed with clear boundaries and straightforward access on CT.	Yes	Yes
Age (y)	Continuous	No	Yes
Sex (female)	Binary: 1 female; 0 male	No	Yes

The primary model intentionally used preoperative covariates available before approach selection. The expanded sensitivity model additionally included age and sex. *PS*, Propensity score; *BMI*, body mass index.

**Table A1a:** Components of the preoperative high-complexity features indicator

Component (pre-incision)	RATS (n = 23), n (%)	VATS (n = 38), n (%)
Any high-complexity feature	3 (13.0)	7 (18.4)
Very large tumor judged difficult to approach/dissect (“tumor overly large”)	3 (13.0)	2 (5.3)
Suspected tight mucosal adherence/indistinct submucosal plane	0 (0.0)	3 (7.9)
Proximal location near the thoracic inlet (≈19 cm from incisors)	0 (0.0)	1 (2.6)
Abutting the aortic arch region (≈24 cm from incisors)	0 (0.0)	1 (2.6)

High-complexity features were adjudicated preoperatively during departmental case discussions by senior attending surgeons together with the operating surgeon, based on pre-incision imaging and endoscopic assessment. Components reflect the recorded special-case reason in the source dataset; tumor size alone was not automatically coded as high complexity. *RATS*, Robotic-assisted thoracic surgery; *VATS*, video-assisted thoracoscopic surgery.

**Table A2:** Weight diagnostics and effective sample size

Weights	Min	99th percentile	Max	Winsorized (capped) proportion	ESS (overall)
Stabilized IPTW (primary)	0.500	2.378	2.508	3.3%	52.2

Stabilized weights were winsorized by capping at the 1st and 99th percentiles before primary IPTW estimation. *ESS*, Effective sample size; *IPTW*, inverse probability of treatment weighting.

**Table A2b:** Stabilized inverse probability of treatment weighting distribution after winsorization, by group

Group	Min	P1	P5	Median	P95	P99	Max
RATS	0.518	0.521	0.532	0.818	1.808	1.931	1.966
VATS	0.680	0.681	0.691	0.878	1.833	2.346	2.378
Overall	0.518	0.526	0.582	0.870	1.809	2.326	2.378

Percentiles describe the stabilized IPTW distribution after capping. RATS, Robotic-assisted thoracic surgery; VATS, video-assisted thoracoscopic surgery.

**Table A3:** Primary inverse probability of treatment weighting treatment-effect estimates (weighted least squares with robust HC1 standard errors)

Outcome	ATE (RATS–VATS)	95% CI	*P* value
PLOS, d	−0.85	−1.53 to −0.16	.015
Total length of stay, d	−0.81	−2.19 to 0.57	.253
Estimated blood loss, mL	−31.51	−41.02 to −22.00	<.001
Operative time, min	−6.76	−11.57 to −1.96	.006
Drain removal time, d	−0.83	−1.30 to −0.35	<.001
Pain duration, d	−1.49	−2.19 to −0.79	<.001
Time to full recovery, d	−6.95	−11.21 to −2.70	.001
Direct in-hospital cost, CNY	15,895	13,126-18,664	<.001

ATE values are mean differences for RATS minus VATS. Negative values favor RATS for duration, blood loss, and time end points; positive values indicate higher direct in-hospital cost with RATS. *ATE*, Average treatment effect; *RATS*, robotic-assisted thoracic surgery; *VATS*, video-assisted thoracoscopic surgery; *PLOS*, postoperative length of stay; *CNY*, Chinese yuan.

**Table A4:** Gamma generalized linear model sensitivity results for skewed outcomes

Outcome	Adjusted mean ratio (RATS/VATS)	95% CI	*P* value
Estimated blood loss, mL	0.372	0.306-0.452	<.001
Direct in-hospital cost, CNY	1.224	1.192-1.257	<.001

Models used a Gamma family with log link and adjusted for BMI, tumor size, tumor location, comorbidity, symptomatic status, High-complexity features, age, and sex. Values are adjusted mean ratios (RATS/VATS). *RATS*, Robotic-assisted thoracic surgery; *VATS*, video-assisted thoracoscopic surgery; *CNY*, Chinese yuan.

**Table A5:** Augmented inverse probability weighting sensitivity results

Outcome	AIPW ATE (RATS–VATS)	Bootstrap 95% CI
PLOS, d	−0.68	−1.09 to −0.27
Total length of stay, d	−0.41	−1.12 to 0.29
Estimated blood loss, mL	−28.92	−34.62 to −23.50
Operative time, min	−7.41	−13.45 to −0.73
Drain removal time, d	−0.67	−1.03 to −0.28
Pain duration, d	−1.30	−1.72 to −0.84
Time to full recovery, d	−6.21	−9.08 to −3.56
Direct in-hospital cost, CNY	16,892	14,933-18,855

AIPW used the primary PS covariates and outcome models fit by treatment arm; CIs were obtained using nonparametric bootstrap resampling. *RATS*, Robotic-assisted thoracic surgery; *VATS*, video-assisted thoracoscopic surgery; *PLOS*, postoperative length of stay; *CNY*, Chinese yuan; *AIPW ATE*, average treatment effect estimated by augmented inverse probability weighting.

**Table A6:** Exploratory propensity score–matching effect estimates

Outcome	Matched pairs	ATE (mean difference)	95% CI	*P* value
Postoperative LOS (d)	16	−0.81	−1.64 to 0.02	.055
Total LOS (d)	16	−0.88	−2.57 to 0.82	.289
Estimated blood loss (mL)	16	−28.62	−40.56 to −16.69	< .001
Operative time (min)	16	−5.38	−9.96 to −0.79	.025
Drain removal (d)	16	−0.88	−1.52 to −0.23	.011
Pain duration (d)	16	−1.12	−2.08 to −0.17	.023
Time to full recovery (d)	16	−5.81	−10.99 to −0.63	.030
Direct in-hospital cost, CNY	16	16,103	12,243-19,963	<.001

One-to-one nearest-neighbor matching without replacement used a caliper of 0.2 SD of the logit PS. Sixteen matched pairs were retained; estimates are exploratory because residual imbalance persisted after matching. *ATE*, Average treatment effect; *LOS*, length of stay; *CNY*, Chinese yuan.

**Table A7:** Multivariable regression with robust HC1 standard errors

Outcome	Adjusted β	95% CI	*P* value
Postoperative LOS (d)	−0.74	−1.27 to −0.20	.007
Total LOS (d)	−0.64	−1.71 to 0.43	.238
EBL (mL)	−31.05	−37.85 to −24.24	<.001
Operative time (min)	−6.17	−10.11 to −2.23	.002
Drain removal (d)	−0.78	−1.22 to −0.34	<.001
Pain duration (d)	−1.52	−2.04 to −1.00	<.001
Time to full recovery (d)	−7.27	−10.59 to −3.94	<.001
Direct in-hospital cost, CNY	15,981	13,650-18,312	<.001

Models adjusted for BMI, tumor location, tumor size, any comorbidity, symptomatic status, and preoperative high-complexity features flag. *LOS*, Length of stay; *EBL*, estimated blood loss; *CNY*, Chinese yuan.

**Table A8:** Alternative winsorization sensitivity results for primary inverse probability of treatment weighting estimates

Winsorization (capping)	Weight cap (min–max)	Outcome	ATE (RATS–VATS)	95% CI	*P* value
1.0th–99.0th pct	0.518-2.378	Postoperative LOS (d)	−0.85	−1.53 to −0.16	.015
1.0th–99.0th pct	0.518-2.378	Estimated blood loss (mL)	−31.51	−41.02 to −22.00	<.001
1.0th–99.0th pct	0.518-2.378	Operative time (min)	−6.76	−11.57 to −1.96	.006
1.0th–99.0th pct	0.518-2.378	Direct in-hospital cost, CNY	15,894.76	13,126.01-18,663.51	<.001
2.5th–97.5th pct	0.537-2.129	Postoperative LOS (d)	−0.84	−1.52 to −0.16	.016
2.5th–97.5th pct	0.537-2.129	Estimated blood loss (mL)	−31.34	−40.81 to −21.88	<.001
2.5th–97.5th pct	0.537-2.129	Operative time (min)	−6.78	−11.60 to −1.96	.006
2.5th–97.5th pct	0.537-2.129	Direct in-hospital cost, CNY	15,902.54	13,126.42-18,678.67	<.001
5.0th–95.0th pct	0.582-1.809	Postoperative LOS (d)	−0.81	−1.49 to −0.14	.017
5.0th–95.0th pct	0.582-1.809	Estimated blood loss (mL)	−30.94	−40.27 to −21.61	<.001
5.0th–95.0th pct	0.582-1.809	Operative time (min)	−6.80	−11.66 to −1.94	.006
5.0th–95.0th pct	0.582-1.809	Direct in-hospital cost, CNY	15,962.24	13,198.78-18,725.70	<.001

Winsorization was implemented by capping stabilized IPTW at the specified percentiles and re-estimating weighted least squares models with HC1 robust standard errors. *RATS*, Robotic-assisted thoracic surgery; *VATS*, video-assisted thoracoscopic surgery; *LOS*, length of stay; *CNY*, Chinese yuan; *ATE*, average treatment effect.

**Table A9:** Primary propensity-score model coefficients

Predictor	β (log-odds)	OR	95% CI (OR)	*P* value
Intercept	−2.680	0.069	0.000-14.890	.329
BMI (kg/m^2^)	−0.036	0.965	0.780-1.192	.739
Tumor location (cm from incisors)	0.060	1.062	0.942-1.197	.323
Tumor size (cm)	0.267	1.306	0.953-1.790	.096
Any comorbidity (yes vs no)	1.373	3.947	0.969-16.079	.055
Symptomatic (yes vs no)	0.095	1.100	0.257-4.702	.898
High-complexity features flag (yes vs no)	−2.177	0.113	0.012-1.098	.060

The model included BMI, tumor location, tumor size, comorbidity, symptomatic status, and high-complexity features flag; AUC = 0.699. *BMI*, Body mass index.

**Table A10:** Operating-room occupancy time sensitivity analysis

Outcome	ATE (RATS–VATS)	95% CI	*P* value
Operative time (skin incision to chest closure; min)	−6.76	−11.57 to −1.96	.006
OR occupancy time (operative time + setup; min)	−1.76	−6.57 to 3.04	.472

OR occupancy time was approximated by adding typical preincision setup times (15 minutes for robotic docking/setup and 10 minutes for thoracoscopic setup) to skin-to-skin operative time. This descriptive analysis contextualizes workflow definitions. *ATE*, Average treatment effect; *RATS*, robotic-assisted thoracic surgery; *VATS*, video-assisted thoracoscopic surgery.

**Table A11:** Calendar-time sensitivity results

Outcome	Adjusted MD (RATS–VATS)	95% CI	*P* value
PLOS, d	−0.75	−1.28 to −0.21	.006
Estimated blood loss, mL	−31.16	−38.01 to −24.32	<.001
Operative time, min	−6.39	−10.06 to −2.73	<.001
Direct in-hospital cost, CNY	15,944	13,593-18,295	<.001

Adjusted mean differences are RATS minus VATS. The direction of PLOS, estimated blood loss, operative time, and cost estimates remained concordant with the primary IPTW analysis after adjustment for surgery year. *RATS*, Robotic-assisted thoracic surgery; *VATS*, video-assisted thoracoscopic surgery; *PLOS*, postoperative length of stay; *CNY*, Chinese yuan.

## Appendix E2 Statistical Appendix

Robotic-Assisted versus Video-Assisted Thoracoscopic Enucleation for Esophageal Leiomyoma.

Study cohort: 61 patients (RATS, n = 23; VATS, n = 38). This appendix documents the prespecified propensity-score workflow, diagnostic checks, and sensitivity analyses supporting the revised manuscript.

A. Analytic cohort, end points, and prespecified coding

Treatment indicator was RATS versus video-assisted thoracoscopic surgery (VATS). The primary end point was PLOS (days). Secondary end points included total length of stay (TLOS), estimated blood loss (EBL), operative time, drain removal time, pain duration, time to full recovery, and direct in-hospital cost (CNY).

Pain duration was defined as days from surgery until no incisional/chest pain requiring analgesics for usual daily activity. Full recovery was defined as return to usual daily activities with tolerance of a normal oral diet. Direct in-hospital cost was defined as the direct hospital charge for the index admission, expressed in CNY; no cost-effectiveness inference was performed.

Baseline covariates and continuous outcomes were complete in the analytic dataset. The High-complexity features field was coded as no when blank, reflecting the source charting structure of positive documentation only.

B. Propensity-score specification and weight diagnostics

The primary propensity-score (PS) model was estimated using logistic regression with clinically prespecified preoperative variables available before approach selection. Stabilized inverse probability of treatment weights (IPTW) were calculated and winsorized by capping at the 1st and 99th percentiles. Model discrimination was modest (primary PS model AUC = 0.699; expanded PS model AUC = 0.715).

C. Primary IPTW estimates

D. Sensitivity analyses

Sensitivity analyses were interpreted as robustness checks rather than independent proof of causality. Discrepant estimates were interpreted cautiously because they reflect different estimates, retained samples, and/or modeling assumptions.

E. Calendar-time sensitivity analysis

To assess potential calendar-time confounding related to learning curve and pathway evolution, multivariable linear regression models additionally adjusted for surgery year as a continuous covariate alongside the primary PS covariates.
